# Sports, in association with specific exercises, can help to achieve better results in controlling the evolution of scoliosis

**DOI:** 10.1186/1748-7161-8-S1-O19

**Published:** 2013-06-03

**Authors:** M Romano, S Negrini

**Affiliations:** 1 ISICO (Italian Scientific Spine Institute) Milan, Italy; 2 University of Brescia, Brescia, Italy; 3 IRCCS Don Gnocchi, Milan, Italy

## Background

SOSORT Guidelines recommend that patients, who follow a conservative treatment program for scoliosis, practice sport activities in association with Specific Physiotherapy Exercises (PSE). From a theoretical point of view, the sport activity combines well with the goals of treatment of a disease characterized by a postural dysfunction.

## Aim

The purpose of this study is to compare the results at the end of rapid growth spurt (Risser 3), between a group of patients treated with a conservative protocol (exercise and/or brace), and a group of patients who have added some sport activity to the same protocol.

## Methods

We evaluated 543 patients (497 females/45 males) treated for idiopathic scoliosis with either PSE only (144 patients, 15.5°±9.3°Cobb), or brace and PSE (399 patients, 33.3°±12.1°). Patients started treatment at Risser 0-1, with a minimum age of 10 years, and were followed up to Risser 3. A comparison was then made between the following subgroups:

PSE + Sport (PSESP: 88 patients, 14.8°±5.7°) vs PSE only (PSE: 56 patients, 16.6°±13.1°)

Brace + PSE + sport (BPSESP: 182 patients, 32.2°±10.7°) vs Brace + PSE (BPSE: 217 patients, 34.2°±13.2°) Outcome: Variation of °Cobb at Risser 3 Statistical analysis: ANOVA, T-Test- -

## Results

At the onset we did not find statistically significant differences between the groups. The comparison of °Cobb at Risser 1, and 3, shows better results in PSESP (improvement of 0.53 °) compared to PSE (progression of 1.75 °), but the difference is not statistically significant. Analysis of the results of braced patients at Risser 3 showed improvement of both groups (BPSESP 3.87°, BPSE 3.01°). The difference for the final result was statistically significant (P=0.04).

## Conclusions

In the context of conservative treatment, sport activity, in association with a specific exercise program, seems to be useful to contrast the evolution of scoliosis, especially for braced patients.
